# Electrochemical fabrication of FeS_*x*_ films with high catalytic activity for oxygen evolution

**DOI:** 10.1039/c9ra05343c

**Published:** 2019-10-09

**Authors:** Wenbin Wang, Ruidong Xu, Bohao Yu, Xuanbin Wang, Suyang Feng

**Affiliations:** Faculty of Metallurgical and Energy Engineering, Kunming University of Science and Technology Kunming 650093 China 2545845040@qq.com; State Key Laboratory of Complex Nonferrous Metal Resources Clean Utilization Kunming 650093 China

## Abstract

Electrochemical decomposition of water to produce oxygen (O_2_) and hydrogen (H_2_) through an anodic oxygen evolution reaction (OER) and a cathodic hydrogen evolution reaction (HER) is a promising green method for sustainable energy supply. Here, we demonstrate that cauliflower-like S-doped iron microsphere films are materials that can efficiently decompose water as an electrocatalyst for the oxygen evolution reaction. FeS_*x*_ films are prepared by a simple one-step electrodeposition method and directly grow on copper foam from a deep eutectic solvent, ethaline (mixture of choline chloride and ethylene glycol), as a durable and highly efficient catalyst for the OER in 1.0 M KOH. The prepared FeS_*x*_/CF, as an oxygen-evolving anode, shows remarkable catalytic performance toward the OER with a moderate Tafel slope of 105 mV dec^−1^, and require an overpotential of only 340 mV to drive a geometrical catalytic current density of 10 mA cm^−2^. In addition, this catalyst also demonstrates strong long-term electrochemical durability. This study provides a simple synthesis route for practical applications of limited transition metal nano catalysts.

## Abstract 2

Electrochemical decomposition of water to produce oxygen (O_2_) and hydrogen (H_2_) through an anodic oxygen evolution reaction (OER) and a cathodic hydrogen evolution reaction (HER) is a promising green method for sustainable energy supply.

## Graphical abstract

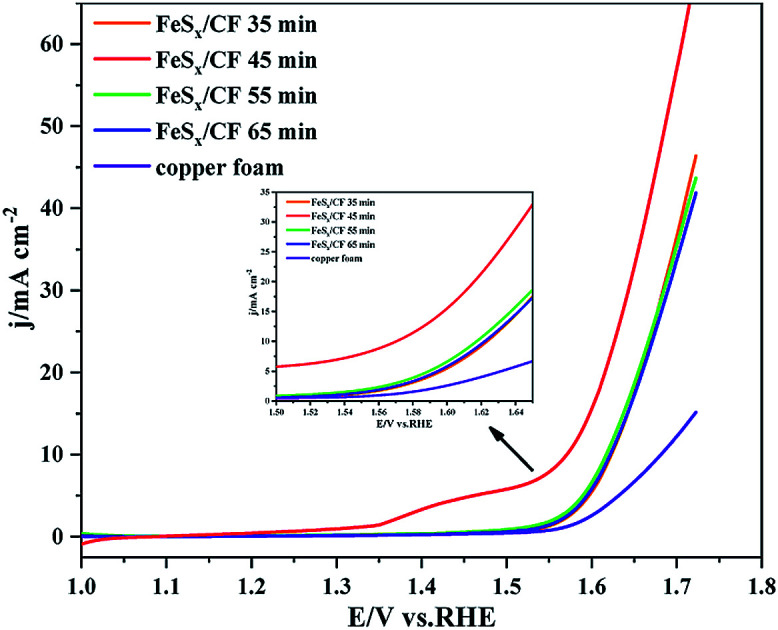

## 1. Introduction

Over the past decades, as the world's population has grown and living standards have changed, energy has gained widespread attention as a fundamental driver.^1,2^ In recent years, researchers have attempted to achieve sustainable development by developing new energy sources and utilizing renewable energy sources. Solar energy, wind energy, tidal energy, geothermal energy and other energy sources are expected to play an important role in the future development of human society. Affected by regional differences and intermittent supply, however, they are difficult to use on a large scale.^3^ To solve the energy and environmental problems, efficient and green energy conversion and storage devices, such as fuel cells,^4^ metal-air batteries^5^ and electrolytic water,^6^ have recently received widespread attention. Among them, electrolytic water is an energy conversion device that converts electric energy into hydrogen energy.^7^ The electrolytic water reaction is carried out in an electrolytic cell. Under the action of direct current and catalyst, the water generates hydrogen and oxygen through electrochemical reaction on the electrode.^8^ The cathode reacts with hydrogen evolution catalyst to produce hydrogen (HER), and the anode reacts with oxygen evolution catalyst to produce oxygen (OER). The oxygen release reaction has a slow kinetics (High Overpotential). Therefore, OER can be performed in any medium, at room temperature (293 K), OER has a thermodynamic potential of 1.23 V *vs.* RHE.^9^ However, in order to accelerate the catalytic reaction, a potential higher than the thermodynamic potential must be applied, which will lead to the consumption of excess energy and reduce the conversion frequency.^10–15^ This extra potential (also called an overpotential) *η* mainly have intrinsic reactive activation barrier and some other resistances, such as solution resistance and contact resistance.^16^ Therefore, it is urgent to develop OER catalysts with high catalytic performance and cost performance.

In recent years, although most non-expensive HER and OER electrocatalysts are based on crystalline compounds, an increasing number of amorphous materials have become more effective electrocatalysts than their crystalline counterparts, mainly including amorphous metal sulfides (*e.g.*, MoS_*x*_ and CoS_*x*_) for HER^17–20^ and amorphous metal oxides (*e.g.*, MO_*x*_, M = Fe, Co, and Ni) for OER.^21–25^ Although some progress has been made in the study of strategies and catalytic properties for the synthesis of amorphous catalytic materials, fine design of microstructure of new amorphous materials and a deep understanding of their catalytic mechanism are necessary, which it is hoped that the newly developed materials will be efficient for HER and OER. To this end, we prepared a copper based material, which is composed of a high conductivity carrier with high surface area and a large amount of activity. We prepared cauliflower-like FeS_*x*_ microsphere films using choline chloride (ChCl)–ethylene glycol (EG)-based deep eutectic solvent (ethaline) as high-performance OER catalyst using a simple potentiostatic electrodeposition method, with no template required.^17,26–28^ Compared with water molecular solutions, the solvent properties of ethaline can provide a favorable chemical environment for producing nanostructured materials with high catalytic activity.^29^

In this paper, we report a simple and efficient method for preparing self-supporting FeS_*x*_ microsphere films grown directly on copper foam (labeled as FeS_*x*_/CF), which is a highly durable and efficient catalyst for OER in alkaline media. The system without precious metal has unprecedented electrocatalytic activity of water oxidation and low potential stability under various conditions.^30^

## 2. Experimental section

### 2.1 Chemicals and materials

Laboratory equipment and chemicals include ethylene glycol (EG, 99%), choline chloride (ChCl, 99%), ferric chloride hexahydrate (FeCl_3_·6H_2_O, 98%), thiourea (CH_4_N_2_S, 99%), potassium hydroxide (KOH, 95%) are purchased from Aladdin Ltd. (Shanghai, China) and used as received. The deep eutectic solvent ethaline is synthesized by stirring the EG and ChCl together with a molar ratio of 1 : 2 at 353 K until a homogeneous liquid mixture is obtained.^31^

### 2.2 Electrochemical preparation of FeS_*x*_/CF films

The CHI760D electrochemical workstation is the main equipment. 400 mM FeCl_3_·6H_2_O and 10 mM thiourea are weighed and placed in 40 ml eutectic solvent and stirred at 333 K. The FeS_*x*_/CF electrodeposition is carried out in a standard three-electrode system, and s-doped iron films are prepared by one-step potentiostatic deposition. Among them, copper foam (the thickness of 1 mm, purity > 99.99%, 2.3 cm^2^) is used as the working electrode, silver wire and platinum column are used as the reference electrode and the counter electrode, respectively. Before each deposition, the copper foam is ultrasonically cleaned in 1% HCl for 10 min, then in anhydrous ethanol for 10 min, ished with distilled water, and finally dried. The electrodeposition is carried out at a fixed potential of −0.95 V *vs.* Ag wire with various charge densities. After deposition, the samples are ished with anhydrous ethanol and deionized water successively and dried with air for further characterization.

### 2.3 Characterization

The morphology and element composition of the deposited samples are characterized by field emission scanning electron microscopy (SEM, Nove NanoSEM 450) equipped with energy dispersive X-ray (EDS) system. X-ray diffraction (XRD) patterns are recorded on a D/max 2200 X-ray diffractometer (XRD, Cu Kα radiation). X-ray photoelectron spectroscopy (XPS) analysis was detected on a PHI 5500 X-ray photoelectron spectroscope.

### 2.4 Electrochemical test

Electrocatalytic experiments are performed using linear sweep voltammetry (LSV), cyclic voltammetry (CV), and electrochemical impedance spectroscopy (EIS) and chronoamperometry. All measurements are performed at ambient temperature in a CHI760D electrochemical workstation with a 50 mL electrolyte (1.0 M KOH). This experiment FeS_*x*_/CF, Hg/HgO and platinum column are respectively used as the working electrode, reference electrode and counter electrode. All the deposited samples were activated prior to any measurements as reported previously. All potentials, measured against a Hg/HgO electrode, are converted to the potential *versus* the reversible hydrogen electrode (RHE) according to *E* (*vs.*RHE) = *E* (*vs.* Hg/HgO) + 0.096 + 0.0591 pH. LSV is measured at the scanning rate of 5 mV s^−1^ in 1.0 M KOH. Electrochemical impedance spectroscopy measurements are performed at 0.75 V (*vs.* RHE) at the same configuration from 100 000 KHz to 0.1 Hz. Electrochemical two-layer capacitances are measured to determine the surface activity areas of the deposited products. For each sample, CV curves in the non-faraday voltage range (0.62–0.72 V *vs.* RHE) at various scan rates ranges from 5 to 200 mV^−1^. The rough stability of the electrocatalyst prepared for FeS_*x*_/CF is measured for 6000 scans by continuous cycle operation (1.0 to 1.7 V *vs.* RHE for the OER, 100 mV^−1^). The timing-current diagram is measured to assess the durability of the catalyst at 1.50 V in 1.0 M KOH solution.

## 3. Results and discussion

**Fig. 1:**
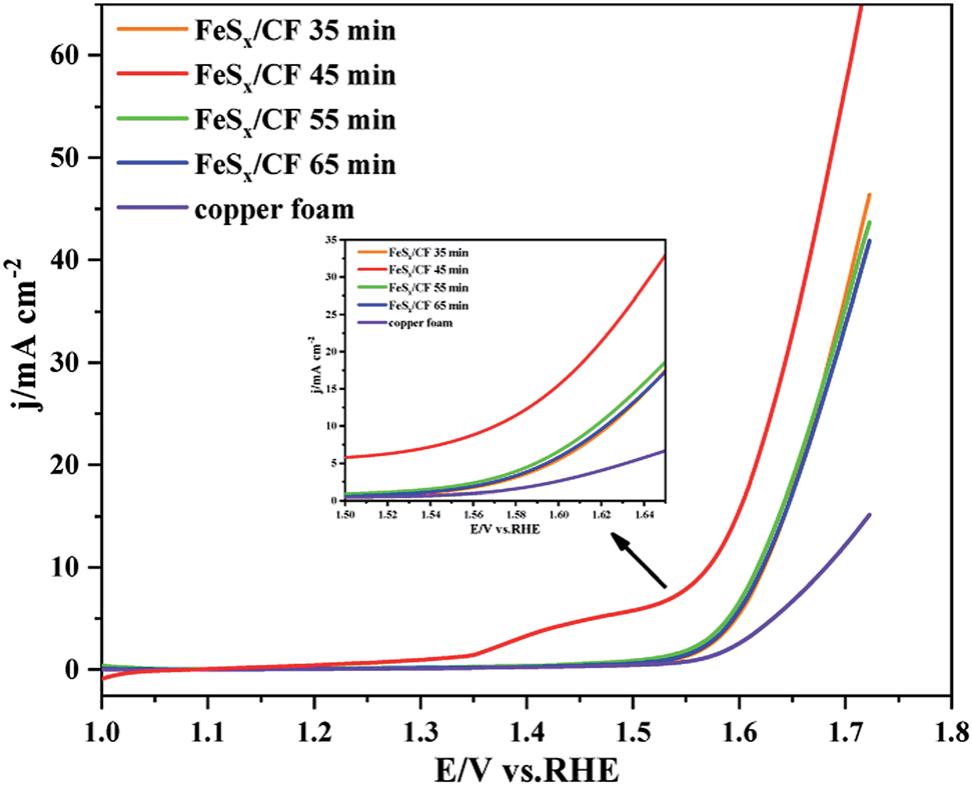
Electrocatalytic water oxidation polarization curves of the FeS_*x*_/CF electrodes fabricated with different electrodeposition time to create the surface FeS_*x*_ species.

Under the condition of 293 K, the voltage of electrolysis at constant potential is −0.95 V (*vs.* Ag wire), and the electrodeposition times are 35 min, 45 min, 55 min and 65 min, respectively. The electrochemical activity for oxygen evolution reaction (OER) was measured by linear sweep voltammetry (LSV) with catalysts obtained at different deposition times. All electrochemical tests were carried out in a solution of 1 M KOH electrolyte, and the LSV shows a scanning rate of 5 mV s^−1^. After the electrodeposition of the copper foam (CF), it is obvious that the fuchsia CF turns black. Fig. 1 shows the polarization curve of electrocatalytic water oxidation of catalyst FeS_*x*_/CF prepared at different electrodeposition times. Comparison of the LSV diagram in Fig. 1 shows that catalyst FeS_*x*_/CF in 1 M KOH solution for electrocatalytic OER, produced by the catalytic current density, increased with the increase of electrodeposition time, and show the trend of initial increase followed by a decrease. When the electrodeposition time increased from 35 min to 45 min, the electrocatalytic activity for OER increased gradually. When the electrodeposition time increased from 45 min to 65 min, electrocatalytic activity for OER gradually decreased. Therefore, it was confirmed that at electrodeposition time of 45 min, the catalytic current density is the maximum at the same voltage as when the obtained catalyst conducts electrocatalytic OER in 1 M KOH solution. In other words, the electrodeposition time of 45 min is the optimal electrodeposition time.

**Fig. 2:**
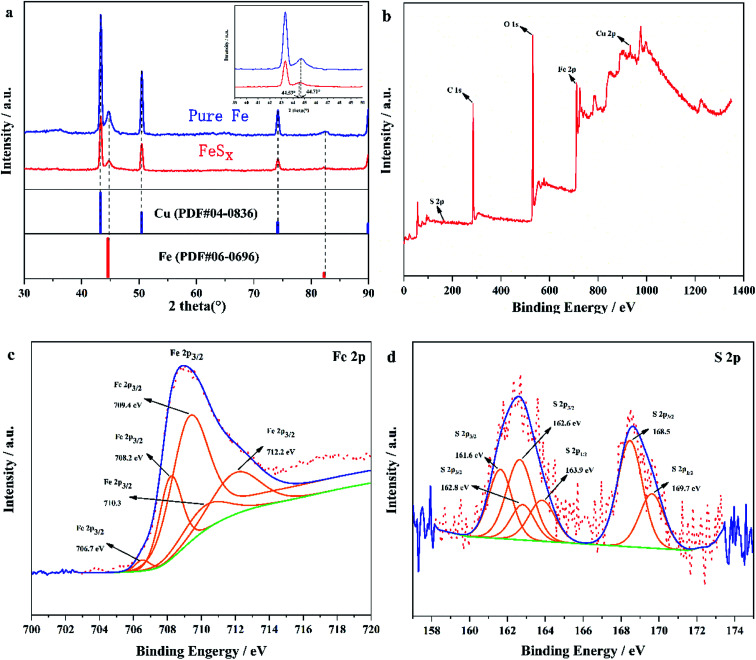
(a) XRD pattern of the sample only consisting of FeS_*x*_/CF. Comparison XPS surveys of FeS_*x*_/CF (b) full survey, (c) Fe 2p and (d) S 2p.

In this experiment, X-ray diffractometer (XRD) was used to analyse catalyst FeS_*x*_/CF prepared with an electrodeposition time of 45 min. Fig. 2a presents the XRD patterns of pure Fe (blue line) and FeS_*x*_ (red line). Except for the strong signal from the Cu substrate, the XRD pattern for the as-prepared Fe/CF (blue line) matches well with the face-centred cubic Fe phase (PDF#06–0696). Diffraction peaks of iron and copper also exist in the samples with added thiourea (red line). This indicates that the deposit containing thiourea in solution is amorphous. In contrast, the S-doped product shows a shift of the diffraction peaks towards lower angles compared with the pure Fe sample (inset in Fig. 2a).

The chemical compositions and surface states of the as-prepared sample were further identified by XPS, as shown in Fig. 2b–d. In the XPS survey, peaks in the full XPS spectrum indicate the existence of Cu, Fe, O, and C elements. The presence of O and C comes from surface attack of the deposited sample and trace solvent residues. XPS spectra of the Fe 2p and S 2p regions of the as-deposited sample are shown in Fig. 2c and d. The Fe 2p levels are split into 2p_3/2_ and 2p_1/2_ doublets owing to the spin–orbit coupling^32–34^ (we only analyze Fe 2p_3/2_ in Fig. 2c). As seen from the XPS image, Fe 2p_3/2_ identified five splitting peaks, and the corresponding binding energies and combined states were 706.7 eV (FeS_2_),^35^ 708.2 eV (Fe_3_O_4_),^36^ 709.4 eV (FeO),^37^ 710.3 eV (FeS),^38^ and 712.2 eV (FeS),^38^ respectively. The two peaks at 708.2 eV and 709.4 eV were attributed to the oxidation of the sample surface. As oxidation is easy when FeS_2_ encounters oxygen, and it can be seen from the XPS spectrum of S that SO_4_^2−^ ions exist at the binding energy 168.5 eV (Fig. 2d). In the iron picture, the binding energy 712.2 eV is attributed to the partial oxidation of FeS_2_ to FeSO_4_.^38^ In the S 2p region (Fig. 2d), the two groups of peak at 161.6 eV, 162.8 eV; and 162.6 eV, 163.9 eV belong to S 2p_3/2_ and S 2p_1/2_ respectively, which are related to the binding energy of S^2−^ and S_2_^2−^ ligands. The other peak at 168.3 eV is the binding energy of Fe–O–S, which is caused by surface oxidation in air.^39,40^ Thus, it suggests that S doped enter into Fe. These results indicate that the FeS_*x*_ obtained by electrodeposition has poor crystal form and may even be amorphous. It also means that the FeS_*x*_ films in FeS_*x*_/CF may consists of small clusters in which Fe is in the FeS and FeS_2_ states. The ratio of the atomic radius of Fe (atomic radius 117 pm) and S (atomic radius 104 pm)^41,42^ is greater than 0.59, which further proves that S is doped into the lattice of Fe to form interstitial compounds. The interstitial compound can be called FeS_*x*_.

**Fig. 3:**
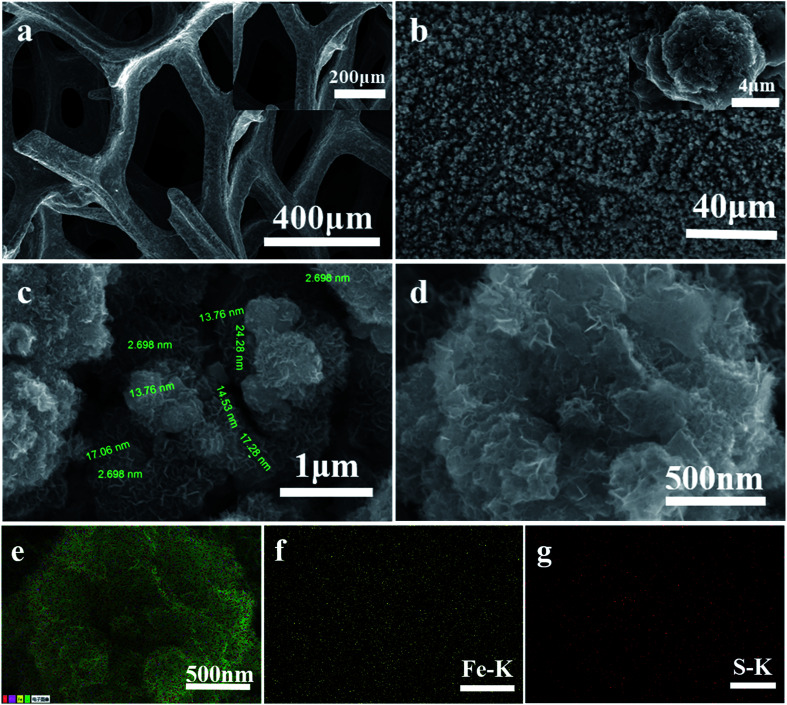
(a) SEM image of copper foam surface. (b–d) The associated high magnification SEM image clearly shows that cauliflower-like FeS_*x*_ microsphere films is made up of many nanosheets. (e–g) The corresponding element mapping images of Fe and S, the scale bars are all 500 nm.

Fig. 3a shows that the whole surface of the CF is bright and smooth. Fig. 3b shows that the whole surface of the CF is uniformly covered with cauliflower-like FeS_*x*_ microsphere films (inset in Fig. 3b). The associated high magnification SEM image (Fig. 3c and d) clearly shows that the cauliflower-like FeS_*x*_ microsphere films is made up of many nanosheets. Meanwhile, samples of the catalyst FeS_*x*_/CF with electrodeposition time of 45 min were characterized by element mapping analysis in SEM. In the element distribution diagrams of SEM, Fe and S are shown in Fig. 3c–e. It can be seen from Fig. 3d and e that Fe and S elements are evenly distributed on the entire sample surface.

**Fig. 4:**
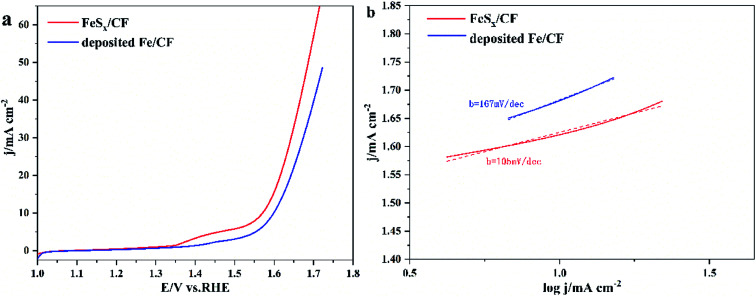
(a) The polarization curves of FeS_*x*_/CF and deposited Fe/CF with scanning rate of 5 mV s^−1^. (b) Corresponding Tafel plots with linear fitting.

The electrocatalytic activity of the prepared FeS_*x*_/CF OER was assessed in 1.0 M KOH by steady-state LSV with a scan rate 5 mV s^−1^. For comparison with the FeS_*x*_/CF catalyst, pure Fe was deposited onto CF electrode by measurement of electrochemical properties. Fig. 4a shows the polarization curve compensation of these catalysts without iR. As expected, FeS_*x*_/CF shows excellent OER activity, whereas the deposited Fe/CF exhibits poor OER activity and greater starting potential. The FeS_*x*_/CF enables OER with high catalytic activity and relatively low starting potential (1.57 V). When the current density reaches 10 mA cm^−2^, the overpotential of the sample in 1 M KOH solution was 340 mV. This overpotential compares favourably with the behaviour of Cu-based OER electrocatalysts, except FeS_*x*_/CF composites such as Fe(OH)_3_ : Cu(OH)_2_/CF (∼365 mV),^43^ Co–CoO@3DHPG (∼410 mV),^44^ FeN_*x*_/carbon (∼320 mV),^45^ NiS/NC (∼371 mV),^46^ and 2D CuO nanosheet bundles (∼350 mV),^47^*etc.* A more detailed comparison is given in Table 1. Fig. 4b shows the Tafel slopes for FeS_*x*_/CF and deposited Fe/CF. The Tafel plots are fitted with the formula: *η* = *b* log *j* + *a* (where *j* is the current density and *b* is the Tafel slope). The Tafel slope of catalyst FeS_*x*_/CF (105 mV dec^−1^) is higher than that of deposited Fe/CF (167 mV dec^−1^), implying more favorable catalytic kinetics on FeS_*x*_/CF.

**Table 1:** Comparison of OER performance for FeS_*x*_/CF and deposited Fe/CF with other nonnoble-metal OER catalysts under alkaline conditions

Catalyst	*j* (mA cm^−2^)	*η* (mV)	Electrolyte	Ref.
FeS_*x*_/CF	10	340	1 M KOH	This work
Deposited Fe/CF	10	369	1 M KOH	This work
Fe(OH)_3_ : Cu(OH)_2_/CF	10	365	1 M KOH	43
Co–CoO@3DHPG	10	410	1 M KOH	44
FeN_*x*_/carbon	10	360	1 M KOH	45
NiS/NC	10	371	1 M KOH	46
2D CuO nanosheet bundles	10	350	1 M KOH	47

**Fig. 5:**
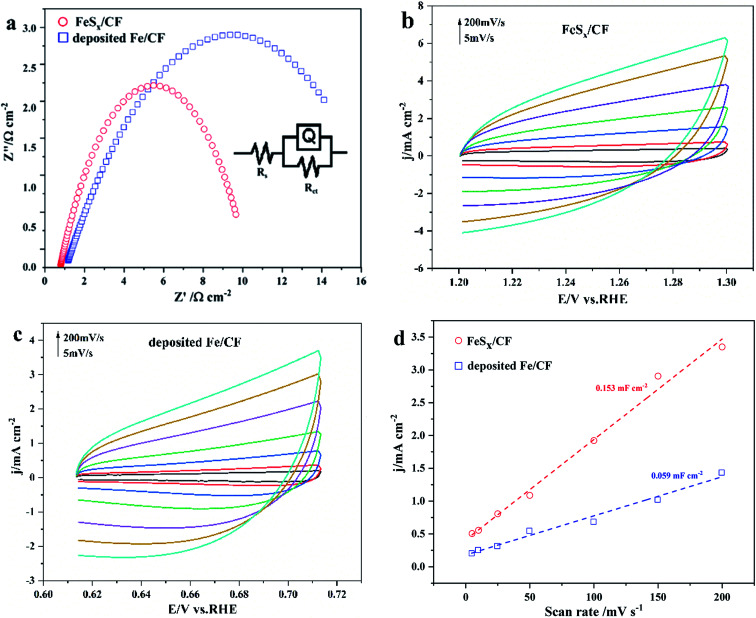
(a) Nyquist plots of FeS_*x*_/CF and deposited Fe/CF at 0.75 V (*vs.* RHE). (b and c) CV curves of FeS_*x*_/CF and deposited Fe/CF catalyst in non-faraday voltage range (0.62–0.72 V *vs.* RHE). (d) The relationship between charging current density difference (*j*_*a*_ − *j*_*c*_) and scanning rate is shown in the figure. The slope in the figure is twice that of *C*_dl_.

Electrochemical impedance spectroscopy (EIS) data (Fig. 5a) reveal that FeS_*x*_/CF, with a much smaller semicircle radius compared with deposited Fe/CF, manifests the high conductivity of the as-prepared electrode, and lower polarization resistance (*R*_ct_). Accordingly, FeS_*x*_/CF has faster charge transfer and OER kinetics. These result show that the electrode dynamics mainly controls the charge transfer process, and the electrochemical system is approximated by the modified Randles circuit, as shown in the inset of Fig. 4a. The potential dependencies of the obtained data include solution resistance (*R*_s_), charge-transfer resistance (*R*_ct_), and constant-phase element related to the double-layer capacitance (CPE).^48^

The contribution of S-doping to the catalytic activity of these electrodes was evaluated, considering that the electrocatalytic activity for OER is highly dependent on their electrochemically active surface area (ECSA) and reaction active sites. CVs of catalyst FeS_*x*_/CF and deposited Fe/CF samples were recorded at different scanning rates from 5 to 200 mV s^−1^ in the faradaic silent region (Fig. 5b and c). From the measured capacitances (Fig. 5d), the *C*_dl_ of porous FeS_*x*_/CF and deposited Fe/CF are 0.153 mF cm^−2^ and 0.059 mF cm^−2^, respectively, suggesting a much larger ECSA of the FeS_*x*_/CF electrode, which is consistent with the LSV and EIS results. The findings suggest that the large ECSA generated by the structural characteristics of FeS_*x*_/CF plays a crucial role in its high catalytic activity. The rough nanosheet structure not only leads to more effective exposure of catalytic activity sites, but also promotes rapid electron transfer and ion diffusion, thereby improving OER catalytic activity.

**Fig. 6:**
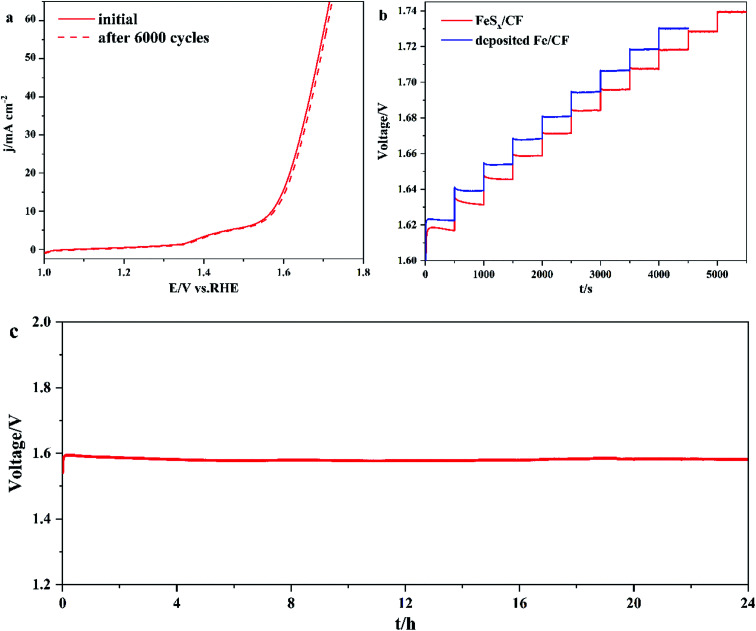
(a) At the scanning rate of 100 mV s^−1^, the polarization curve of FeS_*x*_/CF before and after 6000 voltage sweeps between 1.0 V and 1.7 V *vs.* RHE. (b) Multistep chronopotentiometric curve for FeS_*x*_/CF with current density ranging from 10 to 60 mA cm^−2^, and the deposited Fe/CF with current density ranging from 10 to 50 mA cm^−2^, without iR compensation. (c) Chronopotential curve recorded at constant current density of 10 mA cm^−2^.

In order to obtain the stability of FeS_*x*_/CF, CV scans were performed at 1.0 and 1.7 V *vs.* RHE for 6000 consecutive cycles with a scanning rate of 100 mV s^−1^. The FeS_*x*_/CF catalyst remained stable after thousands of cycles (Fig. 6a). To further prove stability, Fig. 6b shows a multistep chronopotentiometric curve for FeS_*x*_/CF with current density ranging from 10 to 60 mA cm^−2^ (5 mA cm^−2^ per 500 s), and the deposited Fe/CF with current density ranging from 10 to 50 mA cm^−2^ (5 mA cm^−2^ per 500 s). In the range of 1.60–1.74 V, all steps remained unchanged for 500 s, which means that the FeS_*x*_/CF electrode has higher conductivity, better mechanical strength, and better mass transfer than the Fe/CF electrode. For long-term electrolysis at a constant current density of 10 mA cm^−2^ (Fig. 6c), the FeS_*x*_/CF exhibits strong electrochemical stability, maintaining its original OER activity for at least 24 h with negligible degradation. These results show that the FeS_*x*_/CF electrode has a great potential in practical alkaline water electrolysis applications.

## 4. Conclusion

In summary, FeS_*x*_ films is successfully prepared on copper foam by a simple electrodeposition method. The as prepared FeS_*x*_/CF catalyst shows excellent electrocatalytic activity, durability and low overpotential. It is expected that FeS_*x*_/CF hold great promise for developing a cheap electrode material for electrocatalytic OER. Notably, this electrode material has great potential for large-scale and low-cost oxygen production through water decomposition. Besides, the proposed simple electrodeposition method can use to prepare other transition metal based nanomaterials for application of electrochemical oxygen generation.

## Conflicts of interest

There are no conflicts to declare.

## Supplementary Material
